# Swiss Dairy Farmers' Perceptions Surrounding the Disposal of Waste Milk Containing Antibiotic Residues and Antibiotic Resistance

**DOI:** 10.3389/fvets.2021.787828

**Published:** 2022-01-26

**Authors:** Véronique Bernier Gosselin, Vivianne H. M. Visschers, Michèle Bodmer, Mireille Meylan

**Affiliations:** ^1^Clinic for Ruminants, Vetsuisse Faculty, University of Bern, Bern, Switzerland; ^2^School of Applied Psychology, University of Applied Sciences and Arts Northwestern Switzerland, Olten, Switzerland

**Keywords:** non-saleable milk, antimicrobial residues, antimicrobial resistance, risk perception, personal values, behavior, farmers' beliefs

## Abstract

The feeding of waste milk containing antibiotic residues (WMA) to calves has been associated with the shedding of antibiotic-resistant bacteria by calves. However, little is known regarding farmers' intrinsic factors affecting this practice, and on which it would be relevant to intervene in order to change this practice. The objectives of this study were (1) to describe the farmers' intrinsic factors, such as perceived benefits, risks, and social norms related to the feeding of WMA to calves, antibiotic resistance, and antibiotic use, and (2) to evaluate how the feeding of WMA to calves is related to farmers' personal values and perceptions related to WMA feeding and antibiotic resistance. Answers to an online survey were collected from 233 Swiss dairy producers (38.3% response rate). The proportion of respondents who fed WMA to calves was 48.3%. In a hierarchical logistic regression model, only perception factors extracted by factor analysis were associated with the feeding of WMA to calves, namely (in decreasing order of magnitude): farm-level benefits of WMA feeding, the interaction of farm-level benefits with support from governmental authorities, and causes and threats of antibiotic resistance. The results suggest that, in order to reduce the feeding of WMA to calves, communications to dairy producers should focus on changing the perceived benefits of this practice in comparison to those of alternative WMA disposal methods carrying a lower risk of antibiotic resistance. The involvement of veterinarians and governmental authorities in these communications and in supporting producers may increase the successful adoption of alternative WMA disposal methods.

## Introduction

Treatment of lactating dairy cows with most antibiotic drugs results in the production of waste milk containing antibiotic residues (WMA), which must be withheld from sale during the course of treatment and for a withdrawal period thereafter. On dairy farms in numerous countries, it is common practice to feed WMA to dairy calves (1–6). In a recent study in Switzerland, 47.3% of surveyed dairy producers also used this WMA disposal method (7). However, this practice has come under scrutiny, as it has been associated with the selection of antibiotic resistance (AR) in the calves' commensal microbial flora (8, 9). On the other hand, the disposal of WMA with manure or directly on the fields may also contribute to the persistence and spread of AR genes into the farm environment (8, 10). The efficacy of WMA treatment or disposal methods in inactivating antibiotic residues has been reviewed (8, 11), but the implementation of these methods may be hindered by their limited on-farm applicability, high cost, or both. In Switzerland, the use of enzymes to inactivate antibiotic residues in milk is not permitted, and the disposal of WMA in biogas facilities requires important logistics from the producers, while only the feeding to calves allows to use the nutritional value of the milk (12). In order to decrease the impact of feeding WMA to calves on the development of AR on dairy farms, a better understanding of the factors affecting this practice is required.

Extrinsic factors that have been associated with the feeding of WMA to calves include herd size, lactating cow housing type, geographical region, non-organic production, average cow milk production, and average bulk tank milk somatic cell count (4, 5, 7). On the other hand, little is known regarding farmers' intrinsic factors associated with the feeding of WMA to calves. Farmers' motivations may include economic benefits, convenience, difficulties with disposal, or perceived benefits for calf growth (3). An influence of economic motivation was also supported by the volume of WMA produced being one of the most common factors affecting the farmers' decision to feed WMA to calves (7). However, WMA feeding practices were additionally affected by factors such as the age or purpose of the calves to be fed, the number of days elapsed after completion of treatment or the specific antibiotic drug present in the milk, suggesting that other concerns also are at play (7). Furthermore, in the United Kingdom where veterinary drugs can be purchased by farmers not exclusively from their veterinarian but also on internet pharmacies or in agricultural stores (with a veterinary prescription when required), farmers who purchased veterinary drugs from another source than their veterinarian were more likely to feed WMA to bull calves than to “throw it away” (13). The relationship between farmers and their veterinarian may therefore also be of importance.

Over the past decade, social psychological theories have been increasingly used to investigate factors affecting on-farm implementation of recommended herd health practices in various areas, such as prudent antibiotic use (AU) (14–17). The theory of planned behavior postulates that an individual's intention to perform a behavior is affected by attitudes toward the behavior, subjective norms, and perceived behavioral control, the relative importance of which may vary across behaviors (18). In turn, actual behavior is affected by intention and perceived behavioral control (18). Attitudes reflect beliefs regarding positive or negative attributes of the behavior, whereas subjective norms reflect whether important reference groups would approve or disapprove of a behavior (18). Perceived behavioral control relates to the individual's perception of available resources and opportunities to perform a behavior (18). Additionally, these determinants of intention may be influenced by background factors such as personality, personal values, demographic variables, and information sources (19). Personal values previously found relevant to the topics of AU and AR include individualism, altruism, biospherism, and conservatism (20–23). Personal values can also influence trust in social institutions and uptake of information (22, 23), and therefore determine attitudes such as risk perceptions (24). Trust also determines confidence or perceived support (25).

**Figure 1:**
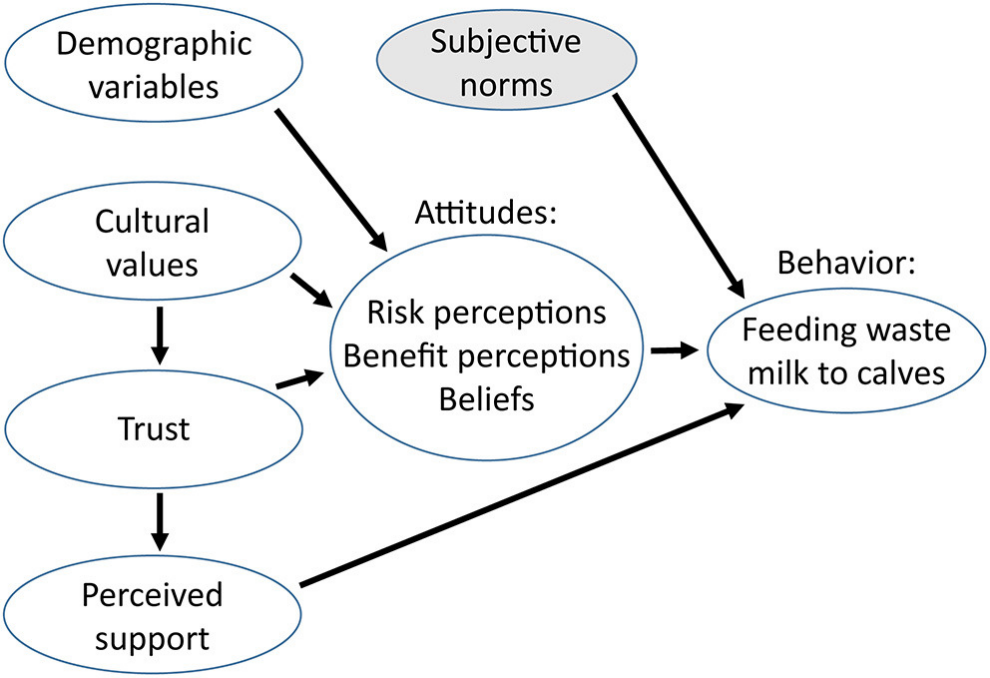
Diagram of the interrelated concepts investigated in relation to the feeding of waste milk containing antimicrobial residues to calves. Subjective norms were not included in the final analyses due to missing data.

A more in-depth understanding of the psychological factors affecting WMA disposal methods on dairy farms is needed, in order to develop interventions that effectively promote WMA disposal practices that would minimize the selection and persistence of AR on these farms. The first objective of this study was to explore the farmers' perceptions related to the feeding of WMA to calves including perceived benefits, risks, social norms, perceived risks of AR, and perceived benefits, social norms, and behavioral control related to AU. Building on the previous questionnaire's findings, perceptions of aggravating factors and of alternative WMA disposal methods were also explored. The second objective was to evaluate the association between the feeding of WMA to calves and selected farmers' personal values and perceptions related to WMA feeding and to AR. The concepts investigated in this study were selected based on the theory of planned behavior, the cultural theory of risk perception, as well as previous studies, and are presented in Figure 1. We therefore hypothesized that the feeding of WMA to calves would be predicted by farmers' attitudes (i.e., perceived benefits, risks and beliefs), social norms, trust in relevant stakeholders, and perceived support. In the present study, perceived behavioral control related to the feeding of WMA to calves was not included because it was considered that producers have full control over their WMA disposal method.

## Methods

A questionnaire was developed to collect information from Swiss dairy producers on their perceptions regarding WMA disposal and AR. The questionnaire was divided into three sections and included a total of 30 questions. Most questions of the first two parts were composed of a partial statement and a number of items to which respondents were asked to indicate their response (generally their level of agreement) on a 6-point Likert scale (Table 1). More specifically, Part 1 included questions on the perceptions related to the feeding of WMA to calves, and grouped based on *a priori* concepts (Table 1), namely risks, benefits for the calves, farm-level benefits (all of these being proxies for attitudes), as well as social norms. Part 1 also included questions on perceived efficacy and costs of alternative WMA disposal methods, willingness to adopt them, other factors affecting the adoption of alternative WMA disposal methods, as well as on trust in and perceived support from stakeholders regarding WMA disposal. Part 2 included questions on the perceptions or beliefs related to causes of AR, threats related to AR, benefits of AU, intention to minimize AU, and social norms regarding AU. Additionally, questions on personal values were included using previously established scale (9-point Likert scale) and items (self-discipline, respect for tradition, humility, respect for the Earth, unity with nature, protection of the environment, equality, helpfulness, family security, and cleanliness) (26). Part 3 included questions on farm characteristics and farmer's demographics.

**Table 1:** Questionnaire items related to perceptions (6-point Likert-scale) and personal values (9-point Likert-scale), with median values and interquartile range (IQR), using responses from 233 dairy producers (unless indicated otherwise, for non-mandatory questions).

**Items^a^**	**Median (IQR)**
Part 1	
1. (Risks) I feel that feeding waste milk containing antimicrobial residues to calves… a. Is safe with regard to calf health. b. Increases the risk of spread of antimicrobial-resistant bacteria.	5 (3–6)^b^ 5 (3–6)
1.2 I feel that the main disposal method I use for waste milk containing antimicrobial residues…	
[Manure pit or manure pile] (*n* = 138)	
a. Is safe with regard to calf health. b. Increases the risk of spread of antimicrobial-resistant bacteria.	2 (1–4)^b^ 3 (2–5)
[On the ground outside] (*n* = 15)	
a. Is safe with regard to calf health. b. Increases the risk of spread of antimicrobial-resistant bacteria.	1 (1–5)^b^ 2 (1–4)
[With wastewater] (*n* = 12)	
a. Is safe with regard to calf health. b. Increases the risk of spread of antimicrobial-resistant bacteria.	1 (1–2.75)^b^ 2.5 (1–3)
[Other] (*n* = 8)	
a. Is safe with regard to calf health. b. Increases the risk of spread of antimicrobial-resistant bacteria.	1.5 (1–3.75)^b^ 2 (1–3.75)
2. (Farm-level benefits) Feeding waste milk containing antimicrobial residues to calves…	
a. Saves a lot of money. b. Saves a lot of time (labor). c. Spares valuable feed. d. Facilitates the daily routine. e. Solves the problem of waste milk disposal. f. Is a convenient disposal method.	3 (1–4) 1 (1–2) 3 (1–4) 2 (1–4) 2 (1–5) 4 (1–5)
3. (Calf-level benefits) I believe that the feeding of waste milk containing antimicrobial residues to calves is associated with…	
a. Better nutritional value of milk. b. Better calf growth.	1 (1) 1 (1)
4. I believe that the risk of bacterial resistance caused by feeding waste milk containing antimicrobial residues to calves increases if…	
a. A critically important (reserve) drug is used. b. The concentration of residues in the milk is high. c. The calves are young. d. The calves are sick. e. The milk also contains bacteria that could cause disease.	5 (4–6) 5 (4–6) 4 (2–6) 4 (2–6) 4 (2–5)
5. The opinion of the following groups of people regarding how I dispose of waste milk containing antimicrobial residues is for me: (1 = not important at all to 6 = very important)	
a. My family b. Befriended milk producers c. My veterinarian d. The dairy company that buys my milk e. My dairy association f. Regulatory authorities (e.g., cantonal/federal veterinary office) g. The general public	5 (4–6; *n* = 231) 4 (3–5; *n* = 231) 5 (4–6; *n* = 230) 5 (2–6; *n =* 231) 3 (1–5; *n =* 231) 4 (3–6; *n =* 231) 5 (3–6; *n =* 231)
6. People who are important to me believe that feeding waste milk containing antimicrobial residues to calves…	
(1 = strongly disagree to 6 = strongly agree; I do not know)	
a. Is the best solution for disposing of waste milk. b. Is dangerous. c. Should be avoided. d. Is acceptable at my farm.	5 (3–6; *n =* 199)^b^ 4 (2–6; *n =* 197) 5 (3–6; *n =* 202) 5 (2–6; *n =* 204)^b^
7. How efficacious do you think the following solutions would be to minimize the effects of antimicrobial residues in waste milk? (1 = not efficacious at all to 6 = extremely efficacious)	
a. Pasteurizing the milk before feeding it to the calves b. Composting the milk with manure c. Collecting the milk for disposal at a specialized facility (e.g., incineration, biogas plant)	2 (1–3) 3 (1–4) 5 (3–6)
8. How costly (i.e., labor and investment) do you think the following solutions would be to minimize the effects of antimicrobial residues in waste milk?	
(1 = not costly at all to 6 = extremely costly)	
a. Pasteurizing the milk before feeding it to the calves b. Composting the milk with manure c. Collecting the milk for disposal at a specialized facility (e.g., incineration, biogas plant)	4 (4–5) 2 (1–3) 6 (5–6)
9. To what extent would you be willing to implement the following solutions to minimize the effects of antimicrobial residues in waste milk (provided they were efficacious)?	
(1 = not willing at all to 6 = extremely willing)	
a. Pasteurizing the milk before feeding it to the calves b. Composting the milk with manure c. Collecting the milk for disposal at a specialized facility (e.g., incineration, biogas plant)	3 (1–4) 5 (2–6) 2 (1–4)
10. If a solution were to be proposed, how would the following aspects influence your willingness to adopt this method? (1 = very negatively to 6 = very positively)	
a. Increased time/labor b. Compensation premium c. Fitting into the daily routine d. Easy to perform e. Impossibility to use the milk as feed f. Benefit to animal health g. Benefit to public health h. Benefit to the environment	5 (4–6)^b^ 4 (3–5) 5 (4–6) 5 (4–6) 2 (1–4)^b^ 6 (5–6) 5 (4–6) 5 (4–6)
11. (Trust) To what extent do you trust the following actors and information sources regarding information about the disposal of waste milk containing antimicrobial residues?	
(1 = do not trust at all to 6 = fully trust)	
a. My veterinarian b. My agricultural/breeding/dairy association c. My dairy company d. Cantonal veterinary authorities e. Federal food safety and veterinary office/Strategy on antibiotic resistance f. Local/national news media	5 (5–6) 4 (3–5; *n =* 230) 4 (3–5; *n =* 232) 4 (3–5; *n =* 232) 4 (3–5; *n =* 232) 2 (1–3)
12. (Support) To what extent do you think that the following actors can support producers, to foster a safer disposal of waste milk containing antimicrobial residues?	
(1 = no support at all to 6 = full support)	
a. My veterinarian b. My agricultural/breeding/dairy association c. My dairy company d. Cantonal veterinary authorities e. Federal food safety and veterinary office/Strategy on antibiotic resistance	5 (3–5) 4 (3–5) 4 (2–5) 4 (3–5) 4 (2–5)
Part 2	
13. (Causes of AR) Antimicrobial resistance may develop in a dairy herd because of…	
a. Too frequent use of antimicrobials. b. Poor selection of antimicrobial treatment for the condition to treat. c. Contact between cattle treated with antimicrobials and other cattle on the farm. d. Introduction of cattle carrying resistant bacteria from other herds. e. Introduction of resistant bacteria from humans visiting or working on the farm. f. Global emergence of resistance, no matter what I do.	5 (4–6) 5 (5–6) 2 (1–4) 5 (4–6) 3 (2–4) 4 (2–5)
14. (Threat of AR) The development of antimicrobial resistance in my herd would represent a threat…	
a. To the health of my cows and calves. b. To animal health in herds of my region. c. To the health of people in contact with my herd (family, workers). d. To human health in the general population. e. To the profitability of my farm. f. To the environment.	6 (5–6) 4 (2–5) 4 (2–5) 4 (2–5) 5 (4–6) 5 (3–6)
15. (Benefit of AU) Please indicate your agreement to each of the following statements.	
a. Antibiotics are easy to apply. b. Antibiotics have a good cost-benefit ratio.	4 (4–6) 4 (3–5)
16. Keeping to a minimum the use of antimicrobial drugs on my farm…	
a. Is desirable. b. Is necessary. c. Is one of my major goals in the short term. d. Is a goal I have been trying to achieve over the past year. e. Is out of my control. f. Is a goal I know how to achieve. g. Is a goal I would need help to achieve. h. Is something I feel responsible for. i. Is an objective that should first be handled by the authorities.	6 (6) 5 (4–6) 5 (4–6) 5 (4–6) 5 (4–6)^b^ 4 (3–5) 3 (2–5)^b^ 6 (5–6) 5 (4–6)^b^
17. People who are important to me…	
(1 = strongly disagree to 6 = strongly agree; I do not know)	
a. Expect me to use antimicrobials prudently. b. Believe antimicrobials should only be applied if absolutely necessary. c. See antimicrobials as an ordinary, unproblematic medicine.	6 (5–6; *n =* 213) 6 (5–6; *n =* 217) 5 (4–6; *n =* 223)^b^
18.How important are the following values as guiding principles in your life? (−1 = opposed to my principles; 0 = not important at all to 7 = extremely important)	
a. Self-discipline: self-restraint, resistance to temptation b. Respect for tradition: preservation of time-honored customs c. Humility: modest, self-effacing d. Respect for the Earth: harmony with other species e. Unity with nature: fitting into nature f. Protection of the environment: preserving nature g. Equality: equal opportunities for all h. Helpfulness: working for the welfare of others i. Family security: safety for loved ones j. Cleanliness: neat and tidy	5 (4–6) 4 (2–5) 5 (4–6) 6 (4–7) 5 (4–6) 6 (5–7) 5 (3–6) 6 (5–6) 7 (6–7) 6 (5–6)

a *Unless indicated otherwise, the scales were: 1 = strongly disagree to 6 = strongly agree*.

b *Responses were reverse-coded before analyses*.

The questionnaire was developed in English, and translated to German and French. The latter two versions were compared by a bilingual co-author to confirm equivalence. Its online format (LimeSurvey, version 2.6.7, Limesurvey, Hamburg, Germany) was then pre-tested by two German-speaking and two French-speaking producers. An invitation to complete the survey was sent electronically to 608 Swiss dairy producers (496 in German and 112 in French) who had participated in a previous survey on WMA management conducted in November-December 2020 (7), and as part of which they had provided an email address to receive invitations to participate in further studies on this topic. The questionnaire was open from March 23rd 2021 to April 21st 2021, and a reminder was sent on April 6th. The objectives of the study were outlined, and the producers were informed that the data would be anonymized and used for scientific purposes only. The producers were further informed that, in order to avoid repetition, their responses to the previous survey, such as herd characteristics, would be matched to their responses to the present survey by use of their email address. Only the first author had access to the original datasets including email addresses. Data retrieved from the previous questionnaire included canton (federal state), organic vs. non-organic production, herd size (number of adult cows), farmer-reported average bulk tank somatic cell count, average cow annual milk yield, and the feeding of WMA produced during the course of treatment, during the withdrawal period following completion of treatment, or both, to calves. With the exception of the question about WMA types fed, questions from the previous questionnaire were not mandatory, therefore some herd characteristics data were missing.

### Statistical Analyses

Completed questionnaire responses were exported from the survey software and analyzed using R version 4.0.3 (27). Responses were inspected for monotony across the questionnaire, range and distribution of answer scores, and missing answers. Cleaned data from the previous questionnaire was merged into the dataset using the producers' email addresses. Descriptive statistics were conducted as proportion of respondents (for categorical data), mean and standard deviation (for normally distributed continuous variables), or median and interquartile range (for non-normally distributed continuous variables and Likert-scale data). Some Likert-scale items were reverse-coded before data analyses to align their meaning with other items (Table 1).

An exploratory factor analysis (EFA) was conducted on 32 perception variables [*psych* package, principal axis method; (28)]. Likert-scale scores were used as continuous variables (29). The pre-analysis concepts included perceived risks (items 1a-b in Table 1), perceived benefits of feeding WMA at the calf-level (item 3b) and at the farm-level (items 2a-f), trust in (items 11a-f) and support from (items 12a-e) stakeholders regarding WMA disposal, and beliefs about the causes (items 13a-f) and threats (items 14a-f) of AR. Item 3a was excluded due to the limited range of respondents' answer scores 1–3. The items intended to assess social norms were not included in the EFA due to a large number of missing answers (*n* = 60). Because personal values items and scales were already established (26), they were analyzed in a separate EFA including 10 variables. Based on the outcomes of the two EFAs, factor scores were obtained for each identified factor, for each producer, and incorporated into the main dataset for further analysis.

A multivariable logistic regression model was built to evaluate the association between WMA feeding and producers' characteristics, factor scores of perceptions, and factor scores of personal values. Herd characteristics that were relevant in previous studies were also evaluated in the model. Independent variables evaluated in the unconditional analyses are presented in Table 2. Variables for which unconditional analysis yielded a *P* < 0.20 were retained for inclusion in the multivariable logistic regression model. In addition, correlation coefficients between retained variables were evaluated and, if applicable, interactions were evaluated in a reduced model. The multivariable logistic regression analysis was built in a stepwise fashion (i.e., hierarchical) by including herd and producer characteristics at step 1, values at step 2, perception factors at step 3, and interactions at step 4, using the Akaike information criterion to check the model fit at each step.

**Table 2:** Independent variables evaluated for inclusion in the multivariable logistic regression model on the probability of feeding waste milk containing antibiotic residues to calves.

**Variable groups**	**Variables**	**Categories (where applicable)**
Herd characteristics	Herd size^a^	< 15; 15–29; 30–44; or ≥ 45 cows
	Bulk tank somatic cell count^a^	< 100,000 cells/ml; 100,000–149,999 cells/ml; or ≥ 150 000 cells/ml
	Average cow annual milk yield^a^	< 6,500 L; 6,500–8,499 L; or ≥ 8,500 L
	Region^a, b^	East (Ostschweiz, Ticino); central (Nordwestschweiz, Zentralschweiz, Zurich); or west (Mittelland, Genferseeregion)
	Production type^a^	Organic; non-organic
	% revenues from milk production	< 25%; 25–50%; 50–75%; > 75%
Farmer's demographics	Gender Age	Male; female
	Highest education level	Secondary or professional training; college or university
	Personnel feeding the calves	The respondent; someone else; variable personnel
Personal value factors	Biospherism Altruism	
	Discipline	
	Tradition	
Perception factors	Causes and threats of AR	
	Trust in and support from other stakeholders	
	Trust in and support from governmental authorities	
	Farm-level benefits of feeding WMA	
	Calf-level benefits of feeding WMA	

a *Data obtained from the previous questionnaire*.

*^b^Federal Statistical Office, 2021 (30)*.

## Results

### Herd and Respondents' Characteristics

A total of 233 dairy producers completed the questionnaire (233/608; 38.3% response rate), including 174 German-speaking and 59 French-speaking producers (response rates: 35.1 and 52.7%, respectively). However, data from the previous questionnaire could only be obtained for 230 producers, and some data from non-mandatory questions were missing. The distribution of farms by herd characteristics is presented in Table 3. The proportion of respondents who feed WMA to calves was 47.6% (111/233). The mean respondents' age was 44.6 years, with a range of 20–63 years (*n* = 232). With the exception of spoken language, the distribution of herd characteristics in the current sample was similar to that of respondents to the previous questionnaire. Accordingly, there was a mild overrepresentation of French-speaking producers and of farms in organic production, and mild underrepresentation of farms from the eastern region of Switzerland, compared to the source population of Swiss dairy farmers (7).

**Table 3:** Distribution of farm characteristics of 233 Swiss dairy producers who completed the questionnaire.

**Herd characteristics (number of responses)**	**Median (interquartile range)**
Number of adult cows (*n =* 227)	28 (20–45)
Average cow annual milk yield (*n =* 224)	7,500 L (6,775–8,500)
	**Number of responses (%)**
Region^a^ (*n =* 210)	
East	39 (18.6%)
Central	54 (25.7%)
West	117 (55.7%)
Production type (*n =* 230)	
Conventional or non-organic label	193 (83.9%)
Organic	37 (16.1%)
Veal calves fattened on the farm (*n =* 230)	
No	155 (67.4%)
Yes	75 (32.6%)
Main WMA^b^ disposal methods used (*n =* 233)	
Manure pit or manure pile	138 (59.2%)
Fed to any calves	33 (14.2%)
Fed to veal calves only	27 (11.6%)
On the ground outside	15 (6.4%)
With wastewater	12 (5.2%)
Other	8 (3.4%)
Person in charge of feeding the calves (*n =* 231)	
The respondent	109 (47.2%)
Someone else	68 (29.4%)
Variable personnel	54 (23.4%)
Proportion of the farm revenues that come from milk production (*n =* 230)	
Less than 25%	6 (2.6%)
25–50%	66 (28.7%)
50–75%	104 (45.2%)
More than 75%	54 (23.5%)
Respondents planning to still be active in milk production	
In five years	182/195 (93.3%)
In ten years	168/195 (86.2%)
In fifteen years	147/202 (72.8%)
Respondent's gender (*n =* 231)	
Male	205 (88.7%)
Female	26 (11.3%)
Respondent's highest level of education (*n =* 231)	
Secondary school	2 (0.9%)
Professional training	189 (81.8%)
College	12 (5.2%)
University	28 (12.1%)

a *East: Ostschweiz, Ticino; central: Nordwestschweiz, Zentralschweiz, Zurich; west: Mittelland, Genferseeregion (30)*.

b *Waste milk containing antimicrobial residues*.

### Perceptions of AR and WMA Feeding

The risks associated with WMA feeding were perceived as high (median score of 5, Table 1, item 1). Producers who did not feed WMA to calves as their main disposal method (*n* = 173) were also asked about the risks of their disposal method. Median risk perception scores were lower than for WMA feeding, although higher for the spread of antibiotic-resistant bacteria than for the risk to calf health (Table 1, item 1.2). The factors most strongly perceived (median score of 5) as increasing the risk of AR caused by WMA feeding were the use of a critically important drug and a high milk concentration of antibiotic residues (Table 1, items 4a,b). When producers were asked about their main source of information regarding AR over the past 6 months (multiple answers allowed), their veterinarian was the main source (71.7%), followed by their agricultural association (26.2%), the national Strategy on antibiotic resistance (16.7%), local or national news (16.3%), cantonal authorities (9.9%), and the Federal food safety and veterinary office (7.7%). To the question about trust in actors and information sources regarding WMA disposal (Table 1, item 11), “other sources” specified included the internet or social media, other media, colleagues, and scientific literature. The trust score for the “other source” item was low (median 2, range 1–3; *n* = 130). Among the perceived threats associated with the development of AR, the items related to the producers' own animals and farm and to the environment yielded the highest scores (Table 1, item 14).

### Exploratory Factor Analyses

The EFA with oblimin rotation with the perception items was conducted on 32 variables and 227 observations. Following an initial analysis, three items were removed because reliability analyses (Cronbach's alpha) on the identified factors revealed poor item-rest correlations for these three variables (items 2f, 3b, and 13f in Table 1). Following a second EFA, two additional items were removed because they had loadings <0.3 on any factor (item 13b), or similar loadings on different factors in the EFA (item 1b in Table 1). The final EFA was conducted on 27 variables. The Kaiser-Meyer-Olkin measure of sampling adequacy was 0.76, and each individual variable's Kaiser-Meyer-Olkin value was > 0.61. Bartlett's test of sphericity showed a significant model, $\chi_{\left(351\right)}^{2}$ = 2,616, *P* < 0.001. Based on the point of inflection on the scree-plot, five factors were retained. Due to correlations between factors, an oblique rotation (i.e., oblimin) was preferred to orthogonal rotation. The factors were interpreted as “causes and threats of AR,” “trust in and support from other stakeholders,” “farm-level benefits,” “support from governmental authorities,” and “trust in governmental authorities,” with proportions of explained variance of 0.12, 0.09, 0.08, 0.07, and 0.06, respectively. Table 4 shows the factor loadings after oblimin rotation, and mean, median, and interquartile range of factor scores. Correlations between factors are shown in Table 5. Based on the median scores, there was a high trust in and support from governmental authorities.

**Table 4:** Summary of the exploratory factor analysis results for the questionnaire on perceptions related to the feeding of waste milk containing antibiotic residues (WMA) to calves and antibiotic resistance (AR), using responses from 227 dairy producers.

		**Oblimin rotated factor loadings**				
**#**	**Items**	**Causes and threats of AR**	**Trust in and support from other stakeholders**	**Farm-level benefits of feeding WMA**	**Support from governmental authorities**	**Trust in governmental authorities**
13e	AR cause: humans visiting or working on the farm	0.56				
14a	AR threat to the health of my herd	0.54				
14b	AR threat to animal health in the region	0.58				
14c	AR threat to health of people in contact with the farm	0.79				
14d	AR threat to human health	0.66				
14f	AR threat to the environment	0.65				
11b	Trust in agricultural association		0.64			
11c	Trust in dairy company		0.71			
12b	Support for safer WMA disposal: agricultural association		0.68			
12c	Support for safer WMA disposal: dairy company		0.78			
2a	Feeding WMA saves money			0.59		
2b	Feeding WMA saves time			0.67		
2c	Feeding WMA spares valuable feed			0.62		
2d	Feeding WMA facilitates daily routine			0.71		
2e	Feeding WMA solves the problem of disposal			0.51		
12d	Support for safer WMA disposal: cantonal authorities				0.79	
12e	Support for safer WMA disposal: FSVO/StAR^a^				0.88	
11d	Trust in cantonal authorities					0.73
11e	Trust in FSVO/StAR^a^					0.69
1a	Feeding WMA is safe with regard to calf health^b^			−0.42		
11a	Trust in veterinarian		0.34			0.44^c^
11f	Trust in local/national news media		0.32			
12a	Support for safer WMA disposal: veterinarian		0.33			
13a	AR cause: too frequent use of antimicrobials	0.31				
13c	AR cause: contact between cattle on the farm	0.50				
13d	AR cause: AR-carrier cattle introduced from other herds	0.47				
14e	AR threat to the profitability of my farm	0.45				
	Eigenvalues	3.36	2.52	2.17	1.79	1.55
	% of variance	0.12	0.09	0.08	0.07	0.06
	Cronbach's α	0.82	0.80	0.77	0.91	0.91^c^
	Mean	0.00	0.00	0.00	0.00	0.00
	Median	−0.009	0.005	−0.08	0.15	0.14
	Interquartile range	−0.72–0.75	−0.71–0.77	−0.76–0.60	−0.80–0.79	−0.60–0.75

a *Federal food safety and veterinary office/Strategy on antibiotic resistance*.

b *Responses were reverse-coded before analyses*.

c *Cronbach's α was calculated excluding the item “Trust in veterinarian”. Only factor loadings > 0.3 are reported*.

**Table 5:** Correlation matrix (Spearman's rho) of personal value and perception variables for inclusion in the multivariable logistic regression model on the probability of feeding waste milk containing antibiotic residues to calves.

**Variable**		**1**	**2**	**3**	**4**	**5**	**6**	**7**
1	Biospherism	1						
2	Altruism	0.65^**^	1					
3	Discipline	0.35^**^	0.45^**^	1				
4	Causes and threats of AR	0.38^**^	0.32^**^	0.24^**^	1			
5	Trust in and support from other stakeholders	0.27^**^	0.21^**^	0.22^**^	0.26^**^	1		
6	Farm-level benefits	−0.32^**^	−0.18^*^	−0.16^*^	−0.24^**^	−0.13	1	
7	Support from governmental authorities	0.09	0.05	−0.06	0.34^**^	0.22^**^	−0.17^*^	1

** *P < 0.01*;

* *P <0.05*.

The EFA with oblimin rotation with the personal values was conducted on 10 variables and 233 observations. Following an initial analysis, one variable was removed due to its loading <0.3 on any factor (item 18i). The Kaiser-Meyer-Olkin measure was 0.83, and each individual variable's Kaiser-Meyer-Olkin value was > 0.77. Bartlett's test of sphericity showed a significant model, $\chi_{\left(36\right)}^{2}$ = 891, *P* < 0.001. Based on the point of inflection on the scree-plot, four factors were retained. Due to correlations between factors, oblique rotation (i.e., oblimin) was preferred to orthogonal rotation. The factors were interpreted as “biospherism,” “discipline,” “altruism,” and “tradition,” with proportions of explained variance of 0.24, 0.14, 0.10, and 0.08, respectively. Table 6 shows the factor loadings after oblimin rotation, and mean, median, and interquartile range of factor scores. Correlations between factors are shown in Table 5. Based on the median scores, the participating producers expressed a high level of biospherism and discipline.

**Table 6:** Summary of the exploratory factor analysis results for the questions related to personal values of 233 dairy producers.

	**Oblimin rotated factor loadings**			
**Items**	**Biospherism**	**Altruism**	**Discipline**	**Tradition**
Respect for the Earth	0.88			
Unity with nature	0.76			
Protecting the environment	0.87			
Equality		0.75		
Helpfulness		0.81		
Cleanliness			0.83	
Humility				0.57
Self-discipline			0.39	
Respect for tradition				0.48
Eigenvalues	2.16	1.28	0.93	0.67
% of variance	0.24	0.14	0.10	0.08
Cronbach's α	0.89	0.77	0.59	0.54
Mean	0.00	0.00	0.00	0.00
Median	0.18	0.12	0.23	0.01
Interquartile range	−0.63–0.76	−0.51–0.67	−0.50–0.71	−0.60–0.75

*Only factor loadings > 0.3 are reported*.

### Associations Between the Feeding of WMA to Calves and Herd Characteristics, Producer's Demographics, Perceptions, and Values

In the unconditional analyses, herd or producer's characteristics associated with the feeding of WMA to calves included region (*P* = 0.10), organic production (*P* = 0.18), average cow annual milk yield category (*P* = 0.03), and gender (*P* = 0.07). Additionally, factors from the two EFA that were associated with the feeding of WMA to calves included the values “biospherism” (*P* < 0.01), “altruism” (*P* = 0.03), and “discipline” (*P* = 0.08), and the perceptions “causes and threats of AR” (*P* < 0.01), “farm-level benefits” (*P* < 0.01), “support from governmental authorities” (*P* < 0.01), and “trust in and support from other stakeholders” (*P* < 0.01). Numerous bivariate correlations between independent variables were detected. Gender was associated with “biospherism” (*P* < 0.01), “altruism” (*P* = 0.01), “causes and threats of AR” (*P* = 0.02), and “support from governmental authorities” (*P* < 0.01). Region was associated with “discipline” (*P* = 0.04), farm-level benefits (*P* < 0.01), and “support from governmental authorities” (*P* = 0.01). Organic production was associated with milk yield category (*P* < 0.01), “biospherism” (*P* < 0.01), “altruism” (*P* = 0.02), and “causes and threats of AR” (*P* = 0.01). Milk yield category was associated with “biospherism” (*P* < 0.01) and “altruism” (*P* = 0.02). Correlations between numerical variables are shown in Table 5. When evaluated in reduced models, interactions were detected between region and “support from governmental authorities” (*P* = 0.03), between “biospherism” and “altruism” (*P* < 0.01), and between “farm-level benefits” and “support from governmental authorities” (*P* < 0.01). At step 1 of the multiple logistic regression analysis, none of the herd and producer characteristics showed a significant association with WMA feeding. The model's explained variance was very low (Table 7). At step 2, the addition of biospherism, altruism, and discipline only mildly improved the model fit and none of the variables showed a significant association with WMA feeding. The addition of the perception factors at step 3 improved the model fit, with “farm-level benefits” and “causes and threats of AR” significantly predicting WMA feeding. At step 4, only the interaction of “support from governmental authorities” with “farm-level benefits” was significant, while the interaction between region and “support from governmental authorities” tended toward significance. The final model's Akaike information criterion was 205 and the model's explained variance (Nagelkerke's R^2^) was 0.54. “Farm-level benefits”, “causes and threats of AR”, and the aforementioned interaction were significantly associated with WMA feeding (Table 7). The most important predictor of feeding WMA to calves was “farm-level benefits.”

**Table 7:** Logistic hierarchical regression model on the probability of feeding waste milk containing antibiotic residues (WMA) to calves (dependent variable), as a function of herd and producer characteristics and factors derived from factor analyses of personal values and perception items related to the feeding of WMA to calves and antibiotic resistance (AR), based on responses from 199 producers (31 missing herd or producer data).

**Effect**	***R*** ^2^ ^*^	**Estimate**	**SE**	***P*** **-value**
Step 1: herd and producer characteristics	0.07			
Intercept		−1.24	0.59	0.04
Producer's gender: male		0.63	0.47	0.18
Central region (reference: west)		0.34	0.36	0.35
Eastern region (reference: west)		0.63	0.40	0.12
Organic production		−0.49	0.44	0.27
Milk yield 6,500–8,499 L (reference: <6,500 L)		0.65	0.43	0.13
Milk yield ≥ 8,500 L (reference: <6,500 L)		0.63	0.47	0.18
Step 2: addition of values	0.13			
Intercept		−0.95	0.62	0.12
Producer's gender: male		0.39	0.49	0.43
Central region (reference: west)		0.41	0.37	0.27
Eastern region (reference: west)		0.74	0.42	0.08
Organic production		−0.37	0.46	0.42
Milk yield 6,500–8,499 L (reference: <6,500 L)		0.53	0.44	0.22
Milk yield ≥ 8,500 L (reference: <6,500 L)		0.45	0.49	0.36
Biospherism		−0.37	0.21	0.09
Altruism		−0.04	0.20	0.86
Discipline		−0.16	0.18	0.37
Step 3: addition of perception factors	0.46			
Intercept		−0.31	0.75	0.68
Producer's gender: male		−0.08	0.58	0.89
Central region (reference: west)		−0.15	0.45	0.73
Eastern region (reference: west)		0.45	0.53	0.39
Organic production		−0.37	0.55	0.50
Milk yield 6,500–8,499 L (reference: <6,500 L)		0.43	0.52	0.41
Milk yield ≥ 8,500 L (reference: <6,500 L)		0.57	0.59	0.33
Biospherism		0.11	0.26	0.67
Altruism		−0.06	0.24	0.80
Discipline		−0.01	0.21	0.95
Causes and threats of AR		−0.55	0.21	<0.01
Farm-level benefits		1.32	0.24	<0.01
Trust in and support from other stakeholders		−0.35	0.19	0.06
Support from governmental authorities		−0.31	0.21	0.13
Step 4: addition of interactions	0.54			
Intercept		−0.21	0.81	0.80
Producer's gender: male		−0.16	0.59	0.79
Central region (reference: west)		0.11	0.51	0.82
Eastern region (reference: west)		0.55	0.58	0.35
Organic production		−0.56	0.62	0.37
Milk yield 6,500–8,499 L (reference: <6,500 L)		0.27	0.57	0.64
Milk yield ≥ 8,500 L (reference: <6,500 L)		0.74	0.65	0.25
Biospherism		0.26	0.30	0.38
Altruism		−0.18	0.28	0.51
Discipline		−0.19	0.23	0.42
Causes and threats of AR		−0.58	0.22	<0.01
Farm-level benefits		1.73	0.32	<0.01
Trust in and support from other stakeholders		−0.37	0.20	0.06
Support from governmental authorities		−0.12	0.32	0.71
Benefits X Support from authorities		−0.84	0.32	<0.01
Central region X Support from authorities		−1.32	0.66	0.05
Eastern region X Support from authorities		−0.40	0.71	0.58

* *Nagelkerke's R^2^*.

## Discussion

The objectives of this study were to describe Swiss dairy farmers' perceptions related to AR and to the practice of feeding WMA to calves, and to evaluate the importance of these perceptions for the use of this WMA disposal practice. Overall, producers were aware of risks associated with the feeding of WMA to calves, and a higher risk perception of AR was associated with a lower probability of feeding WMA to calves. The results therefore suggest that raising producers' awareness about the risks of this practice in relation to AR could be a useful intervention method. Additionally, the characteristics of the residues in milk, such as specific drug or concentration, were perceived to affect the risk of AR associated with this practice. This could explain the previous finding that specific drug received by the cow, time elapsed after treatment, and administration route are producers-reported criteria influencing whether they feed WMA to calves (7). In the study reported here, producers using another main disposal method for WMA than feeding it to calves perceived that the method they use may also carry to some extent a risk for the spread of AR. Consequently, the conceptualization of alternative treatment or disposal methods for WMA could be of potential interest for all dairy producers, and not exclusively for those currently feeding WMA to calves. The perceived threats associated with the development of AR yielded higher scores for items related to the producers' own farm and to the environment, and lower scores for items related to human health, similar to findings of previous reports from the United States (31, 32).

Conversely, the factor “farm-level benefits” of feeding WMA to calves obtained a lower median score than the factor “causes and threats of AR.” Among individual items related to the benefits of feeding WMA, convenience had the highest median score (although not retained in the final EFA due to poor fit), and had previously been identified as one of the main reasons for this disposal practice (13). Along the same lines in the study reported here, among the proposed criteria affecting the willingness to adopt an alternative WMA disposal method, items related to time or labor, easiness to perform, and fitting into the daily routine had high median scores. Although the concern of avoiding feed waste had been raised by a number of dairy farmers in a previous survey on WMA disposal (7), the possibility to use the milk as feed was the only criterion that obtained a low median score. These findings should be considered in the conceptualization and proposal of alternative WMA disposal methods. Disposal of WMA with manure is the most commonly used alternative disposal method (7), and could be considered as a cheap and convenient method, although it may carry a risk for the spread of antibiotic residues and antibiotic-resistant bacteria in the environment (8, 10). However, depending on conditions and management, manure composts have the ability to mitigate, to varying degrees, antibiotic residues, antibiotic-resistant bacteria, and AR genes (11). Among the alternative disposal methods proposed in the present study, composting of milk with manure was the method with the lowest median score of perceived cost and highest median score for willingness to implement by the participants. In addition to promoting herd health and prudent AU, offering trainings to producers for the optimization of composting conditions may be part of the solutions to mitigate the spread of AR in the environment. In order to reduce the feeding of WMA to calves and increase the adoption of alternative methods, proposed alternatives should have similar or improved benefits while presenting a lower risk with regard to the development and spread of AR.

The attending veterinarian was the most common source of information about AR, most trusted information source and most supportive actor regarding safe WMA disposal, and obtained among the highest scores with regard to the importance of their opinion on WMA disposal method. This is consistent with findings of previous reports from the United Kingdom, United States, and Switzerland (14, 16, 33), and emphasizes the role of this group of professionals in disseminating information and counseling producers. Interestingly, items related to trust in and support from federal and cantonal veterinary authorities for the safer disposal of WMA clustered in separate factors, with higher median scores than the factor containing the same items relative to other stakeholders (veterinarian, agricultural association, and dairy company). Wemette et al. (31) reported on concerns from New York State dairy farmers regarding increasing regulations on AU. Similarly, Kramer et al. (17) reported a positive association between AU and a disregard of Dutch farmers for antimicrobial regulations. The results of the present study are not in support of similar concerns or mistrust among Swiss dairy producers toward governmental authorities. Moreover, trust in information sources with regard to WMA disposal could have an additional indirect effect on the practice of feeding WMA to calves, by influencing the perception of risks and benefits of this practice (34). This is further supported by the significant interaction of “support from governmental authorities” with “benefits” observed in the present study. Creating and keeping a trustworthy relationship between governmental authorities, attending veterinarians, and dairy farmers likely plays an important role in ensuring the safe disposal of WMA on dairy farms. Median scores of items related to intention and perceived responsibility to keep AU to a minimum were high, whereas scores of items related to perceived control were lower. Similarly, Jones et al. (14) reported a higher proportion of respondents agreeing that reducing AU is desirable, than the proportion of respondents perceiving having the skills and knowledge to do so. This would suggest that education and support should continue to be offered to assist producers in achieving this goal.

In our previous study, farm characteristics associated with the feeding of WMA to calves in a multivariable logistic regression model included non-organic production, region, herd average somatic cell count, and average cow milk yield (7). However, in the present study where perception factors were also considered, none of the farm characteristics were significant in the final model, with the exception of a tendency toward an interaction effect of region and “support from governmental authorities.” One significant implication of this finding is that WMA disposal practices may be more easily improved by altering perceptions (e.g., through education), whereas extrinsic factors such as production type or geographical location can hardly be changed. Perceived farm-level benefits were the most important factor in the model. This might be explained by these benefits being more tangible in the short term, in comparison to some risks that might be perceived as physically or timely distant or both, such as threat of AR to human health (22). This should be taken into consideration and emphasized in the context of educational interventions. Although some concepts from the theory of planned behavior were used as a basis for this study, the theory was not used *per se*, as some of its components were missing and believed to be irrelevant for the study. Perceived behavioral control was not included because WMA must be disposed of in any case. However, perceived behavioral control may have varied depending on disposal methods available and may have affected their use, for instance with regard to the access to biogas facilities. Additionally, although some of the perceived benefits items may appear to reflect perceived behavioral control, the results of the EFA show that these items did not form a separate construct, such as perceived behavioral control. Subjective norms were excluded from the analyses due to missing answers, presumably because producers may not discuss the topic of WMA disposal with others. Among evaluated personal values, none remained associated with WMA feeding in the final model. However, their associations with farm characteristics and risk and benefit perceptions suggest that they may have an indirect relationship with WMA feeding. This is in line with a report of an indirect relationship of values with AU (20). Trust in information sources and personal values should also be taken into account in future communication strategies of governmental authorities (22).

In this study, the classification of producers into the two WMA feeding categories was based on the respondents' own answer, which may have resulted in a classification bias due to *bona fide* error. Indeed, the previous study revealed that many producers only feed the WMA produced after a number of days into the withdrawal period to calves (7). These producers were categorized as feeding WMA in the previous study, but might perceive that the waste milk they feed contains negligible amounts of residues and therefore, in the questionnaire of the present study, could have stated that they do not feed WMA to calves. However, the impact of this possible bias in the scope of the present study is limited, since their perception of their disposal practice (i.e., intention) is considered more relevant than whether or not the waste milk fed actually contains antibiotic residues.

This study analyzed data from a sizeable sample of Swiss milk producers, allowing EFA to be conducted. Some aspects of the present study, such as perceptions about AR and AU, had previously been studied in association with an intention [e.g., reduced or prudent AU (14, 16)]. However, a strength of the current study is that the perceptions were used to predict a specific behavior in a logistic regression analysis, namely the feeding of WMA to calves. Intentions do not necessarily translate into behavior, and may be biased by social desirability (14, 16). In a previous study, only “knowledge” on antibiotics and AR was significantly associated with the AU behavior measured as defined daily dose, with an explained variance of 0.05 (17). In contrast, the perceptions evaluated in the present study accounted for a large proportion of the variance of the model. Finally, participants were selected based on their participation to a previous survey on WMA disposal, which might have resulted in a selection bias toward a higher level of concern over this topic and the topic of AR (7). Similarly, any bias among the participants to the previous survey with regard to geographical location and organic production would have been carried on in our sample. However, the distribution of farms in our sample suggests that it was overall representative of the general population of dairy farms in Switzerland, and that the results could be generalized to this population (35).

To conclude, milk producers participating to this study did perceive that there are risks associated with the feeding of WMA to calves and with the development of AR in general. The results of the present study show that the perceptions of benefits of WMA feeding, of causes and threats of AR, and support from governmental authorities play an important role in the use of this WMA disposal practice on Swiss dairy farms. Education and eventual intervention strategies should focus on maintaining trust between producers and stakeholders, and counterbalancing the perceived benefits of this WMA disposal practice.

## Data Availability Statement

The raw data supporting the conclusions of this article will be made available by the authors, without undue reservation.

## Author Contributions

VBG performed the data collection and wrote the first draft of the manuscript, and conducted data analyses with support from VHMV. All authors contributed to the design of the study and contributed to manuscript revision.

## Funding

This research was funded by the Clinic for Ruminants, Vetsuisse Faculty, University of Bern, Bern, Switzerland.

## Conflict of Interest

The authors declare that the research was conducted in the absence of any commercial or financial relationships that could be construed as a potential conflict of interest.

## Publisher's Note

All claims expressed in this article are solely those of the authors and do not necessarily represent those of their affiliated organizations, or those of the publisher, the editors and the reviewers. Any product that may be evaluated in this article, or claim that may be made by its manufacturer, is not guaranteed or endorsed by the publisher.

## Abbreviations

AR: Antibiotic resistance
AU: Antibiotic use
EFA: Exploratory factor analysis
WMA: Waste milk containing antibiotic residues.
